# 4D-DIA-Based Quantitative Proteomic Analysis Reveals the Involvement of TRPV2 Protein in Duck Tembusu Virus Replication

**DOI:** 10.3390/v16121831

**Published:** 2024-11-26

**Authors:** Jimin Chen, Fan Yang, Lianjie Lai, Huihuang Li, Chengfu Pan, Xinguo Bao, Weimin Lin, Ruiyi Lin

**Affiliations:** College of Animal Sciences, Fujian Agriculture and Forestry University, Fuzhou 350002, China; jmchen@fafu.edu.cn (J.C.); 12306009008@fafu.edu.cn (F.Y.); 5220609010@fafu.edu.cn (L.L.); lihuihuang@fafu.edu.cn (H.L.); 52306009028@fafu.edu.cn (C.P.); 52306009010@fafu.edu.cn (X.B.); weiminlin@fafu.edu.cn (W.L.)

**Keywords:** duck Tembusu virus, quantitative proteomics, DF-1 cells, TRPV2

## Abstract

Duck Tembusu virus (DTMUV), a novel positive-sense RNA virus, has caused significant economic losses in the poultry industry of Eastern and Southeast Asia since its outbreak in 2010. Furthermore, the rapid transmission and potential zoonotic nature of DTMUV pose a threat to public health safety. In this study, a 4D-DIA quantitative proteomics approach was employed to identify differentially expressed cellular proteins in DTMUV-infected DF-1 cells, which are routinely used for virus isolation and identification for DTMUV, as well as the development of vaccines against other poultry viruses. One hundred fifty-seven differentially expressed cellular proteins were identified, including 84 upregulated and 73 downregulated proteins at 48 h post-infection, among which CXCL8, DDX3X, and TRPV2 may play crucial roles in viral propagation. Notably, for the upregulated protein TRPV2, the DTMUV replication was inhibited in TRPV2-low-expressing DF-1 cells. In summary, our research represents the application of 4D-DIA quantitative proteomics to analyze the proteomic landscape of DTMUV-infected poultry cells. These findings may provide valuable insights into understanding the interaction mechanism between DTMUV and poultry cells, as well as the identification of disease-resistant host factors in poultry breeding research.

## 1. Introduction

Duck Tembusu Virus (DTMUV), belonging to the Orthoflavivirus within the Flaviviridae family, is an emerging RNA virus that belongs to the TMUV group. In recent years, TMUV infections have been reported in poultry in several Asian countries, including China [1,2], Vietnam [3], Thailand [4], and Malaysia [5]. This infection triggers a drastic decline in egg production among egg-laying ducks and neurological impairments in ducklings, with severe cases leading to mortality within flocks. The host range of DTMUV has broadened, extending beyond ducks to encompass geese, chickens, pigeons, sparrows, and even penguins, from which pathogenic DTMUV strains have been isolated [6,7,8]. In particular, DTMUV infection can cause encephalitis and growth retardation in chickens or precipitate Egg Drop Syndrome (EDS) in egg-laying hens [9,10]. DTMUV is capable of stably replicating in chicken embryo fibroblasts (DF-1) [11], yet to date, the proteomic information regarding the avian host cellular response to DTMUV infection remains scarce.

The integration of mass spectrometry-based proteomics with traditional virological research methodologies holds immense potential in unraveling the interactions, localization, differential expression, and functions of viral and host proteins [12]. This, in turn, facilitates a deeper understanding of the intricate relationship and specific mechanisms underlying viral replication and the host’s immune response. Given that viruses rely on cellular organelles and enzymes within living cells for their replication, this invasion triggers an immune response in host cells, leading to alterations in the expression of host proteins. Remarkably, such changes can be detected by various mass spectrometry-based proteomics technologies, thereby facilitating in-depth investigations into virus–host interactions and the discovery of potential antiviral host factors for viruses such as SARS-CoV-2 [13,14,15], influenza A virus (IAV) [16,17], lymphocytic choriomeningitis virus (LCMV) [18,19], herpes simplex virus type 1 (HSV-1) [20,21], and porcine reproductive and respiratory syndrome virus (PRRSV) [22,23]. Among these proteomics technologies, data-independent acquisition (DIA) emerges as a novel mass spectrometry approach that surpasses traditional data-dependent acquisition (DDA) in terms of acquisition efficiency, reproducibility, and accuracy [24]. Furthermore, the advent of four-dimensional (4D) proteomics, which augment the conventional three-dimensional (3D) proteomics framework (comprising retention time, mass-to-charge ratio m/z, and ion intensity) with the additional dimension of ion mobility, offers superior scanning speed and sensitivity. By combining the strengths of 4D proteomics with DIA analysis, the 4D-DIA quantitative proteomics technology comprehensively enhances data integrity while elevating detection sensitivity and throughput [25]. In recent years, the 4D-DIA technology has gradually gained prominence in the precise identification of diagnostic biomarkers, underscoring its potential as a powerful tool for elucidating virus–host interactions and identifying potential antiviral host factors [26,27]. This technology will revolutionize our understanding of viral pathogenesis and host defense mechanisms, paving the way for novel therapeutic strategies and interventions.

In this research, we utilized the state-of-the-art 4D-DIA methodology in conjunction with liquid chromatography–tandem mass spectrometry (LC-MS/MS) to identify and analyze the alterations in host protein expression profiles 48 h post-infection with DTMUV in DF-1 cells. This analysis yielded a total of 157 differentially expressed proteins, with 84 proteins exhibiting significant upregulation and 73 demonstrating notable downregulation in comparison to the uninfected control cells. The in-depth analysis of these proteins offers profound insights into the pathogenesis and immune response dynamics of DTMUV infection, thus furnishing crucial information on potential host antiviral targets that could inform the development of DTMUV-resistant animal breeding strategies.

## 2. Materials and Methods

### 2.1. Cell Culture and Blind Passage of Virus

DF-1 cells are maintained in RPMI 1640 medium (Gibco, New York City, NY, USA) supplemented with 10% fetal bovine serum (CELLiGENT, Hamilton, New Zealand) and incubated under conditions of 39 °C/5% CO_2_. The TMUV strain FQ-C1 (GenBank accession number: KX977555.1) was kindly provided by Dr. Yu Huang from the Fujian Academy of Agricultural Sciences. The virus was propagated in DF-1 cells, and its titer was determined to be 3.8 log10TCID_50_/0.1 mL using the Reed-Muench method [11].

### 2.2. Virus Inoculation

Monolayers of DF-1 cells were cultured in 100 mm cell culture dishes to 80% confluence. The cells were washed twice with a sterile PBS solution (HyClone, Logan, UT, USA), followed by the inoculation of the DTMUV virus at a multiplicity of infection (m.o.i.) of 0.1 TCID_50_/cell. The inoculated cells were incubated at 39 °C with 5% CO_2_ for 2 h, during which time the dishes were gently agitated every 30 min. Subsequently, the cells were washed three times with RPMI 1640 medium and maintained in RPMI 1640 supplemented with 5% fetal bovine serum. Uninfected DF-1 cells served as mock-infected controls. At 48 h post-infection (hpi), both DTMUV-infected and mock-infected cells were collected. Each group underwent three independent biological replicates.

### 2.3. Protein Extraction, Digestion, and Peptide Desalting

For each of the three independent biological replicates of DTMUV-infected and mock-infected DF-1 cell samples, cells were collected using trypsin digestion and resuspended in 2 mM EDTA lysis buffer (China National Medicines Corporation Ltd., Beijing, China) containing 8 M urea and 1 mM PMSF (Xiya Reagent, Linyi, China). The cells were then sonicated on ice for 5 min to disrupt them. Cellular debris was removed from the lysate by centrifugation (15,000× *g*, 10 min, 4 °C), and the supernatant, containing the extracted protein solution, was collected. The total protein concentration was determined using the BCA method (Beyotime Biotechnology, Shanghai, China). Based on the protein concentration, an equal amount of 200 μg of protein solution was taken, and the volume was adjusted to 200 μL with 8 M urea. The proteins were then reduced with 10 mM DTT for 45 min at 37 °C, followed by alkylation with 50 mM iodoacetamide (IAM) for 15 min in the dark at room temperature. Cold acetone (four times the volume of the protein solution) was added, and the mixture was incubated at −20 °C for 2 h to precipitate the proteins. After centrifugation, the protein precipitates were air-dried and resuspended in 200 μL of 25 mM ammonium bicarbonate solution (Xiya Reagent, China) containing 3 μL of trypsin (Promega, Madison, WI, USA). Digestion was performed overnight at 37 °C. Following digestion, peptides from each sample were desalted on C18 column material, concentrated by vacuum centrifugation, and redissolved in 0.1% (*v/v*) formic acid.

### 2.4. LC-MS/MS Analysis

The experimental samples were separated using a NanoElute UHPLC system operating at nanoliter flow rates. The mobile phase consisted of solution A, which was 0.1% formic acid in water, and solution B, which was 100% acetonitrile containing 0.1% formic acid. A total of 200 ng of each sample was injected into an analytical column (IonOpticks, Melbourne, Australia, 25 cm × 75 μm, C18 packing material, 1.6 μm particle size). The analytical column was maintained at 50 °C, and the flow rate was set to 300 nL/min with a gradient elution program spanning 40 min. The liquid chromatography gradient was as follows: 0–25 min, a linear gradient of 2–22% B; 25–30 min, a linear gradient of 22–35% B; 30–35 min, a linear gradient of 35–80% B; and 35–40 min, maintenance of 80% B.

After chromatographic separation of the mixed samples, mass spectrometry data were initially acquired using the timsTOF Pro2 mass spectrometer in the ddaPASEF mode to establish appropriate acquisition windows for the diaPASEF acquisition method. The analysis was performed over an effective gradient of 40 min in positive ion mode, with a parent ion scan range of 100–1700 m/z. The ion mobility 1/K0 range was set to 0.85–1.3 Vs/cm^2^, and the ion accumulation and release time was 100 ms, resulting in nearly 100% ion utilization. The capillary voltage was 1500 V, the drying gas flow rate was 3 L/min, and the drying temperature was 180 °C. For the ddaPASEF acquisition mode, the following parameters were used: 4 MS/MS scans (total cycle time of 0.53 s), charge range of 0–5, dynamic exclusion time of 0.4 min, ion target intensity of 10,000, ion intensity threshold of 1500, and collision energy increasing linearly with ion mobility, starting from 27 eV at 1/K0 of 0.85 V s/cm^2^ to 45 eV at 1/K0 of 1.3. The quadrupole isolation width was set to 2 Th for m/z < 700 and 3 Th for m/z > 800. For the diaPASEF acquisition mode, the parameters were as follows: mass range at approximately 400–1200, mobility range at 0.85–1.3 Vs/cm^2^, mass width at 25 Da, mass overlap at 0.1, mass steps per cycle at 24, and number of mobility windows at 2, resulting in a total of 48 acquisition windows. The average acquisition cycle time was 1.17 s.

### 2.5. Data Analysis

Protein identification was conducted using the DIA-NN software (version 1.8.1) through a library-free approach for database searching, employing the uniprot_UPO00000539_chicken_20230329.fasta database (containing 43,710 sequences in total). Leveraging the spectral library generated based on deep learning parameters and MBR, we re-analyzed the DIA data to obtain protein quantification. Following protein quantification by the search software, sample normalization was performed to facilitate differential quantitative analysis of proteins. To identify differentially expressed proteins between the DTMUV-infected group and the mock-infected group, we calculated the fold change (FC) as the mean ratio of each protein’s quantitative values across all biological replicates. Subsequently, a *t*-test was applied to the quantitative values of each protein between the two groups, and the corresponding *p*-value was computed for statistical significance testing. Proteins with FC > 1.5 or FC ≤ 0.6667 and a *p*-value < 0.05 were defined as significantly differentially expressed proteins.

### 2.6. Bioinformatics Analysis

Bioinformatics analysis of the identified differentially expressed proteins was performed using the Uniprot database and R software (version 4.4.1). This included gene ontology (GO) analysis based on the Gene Ontology platform (http://geneontology.org, accessed on 15 March 2024) and KEGG pathway analysis utilizing the KEGG Mapper platform (http://www.genome.jp/kegg/mapper.html, accessed on 16 March 2024).

### 2.7. RNA Interference

Three chicken-derived TRPV2 small interfering RNAs (TRPV2-siRNAs) and one control small interfering RNA (NC-siRNA) were synthesized by AuGCT Biotech (Beijing, China) (Supporting Information Table S1). TRPV2-siRNA or NC-siRNA was transfected into DF-1 cells grown to approximately 80% confluence in 12-well plates using jetPRIME lipid transfection reagent (Polyplus, France). After 72 h of transfection, the most efficient TRPV2-siRNA was selected for subsequent experiments. Similarly, to validate the effect of knocking down *TRPV2* gene expression on DTMUV replication, DF-1 cells were transfected with the efficient TRPV2-siRNA or NC-siRNA for 24 h, followed by DTMUV inoculation. Cell samples and supernatants were then analyzed 48 h post-inoculation.

### 2.8. RT-qPCR

Total RNA was extracted from DF-1 cells of the DTMUV-infected cells and the mock-infected group using TRIzol (Invitrogen, Carlsbad, CA, USA). Subsequently, 1 μg of total RNA was reverse-transcribed using 2×NovoScript^®^ Plus 1st Strand cDNA Synthesis SuperMix (Novoprotein, Suzhou, China), which eliminates genomic DNA contamination and simultaneously synthesizes the first-strand cDNA. RT-qPCR was performed using NovoStart^®^ SYBR qPCR SuperMix Plus (Novoprotein, China) and specific primers (Table 1). The amplification reaction was programmed as follows: initial denaturation at 95 °C for 1 min, followed by 40 cycles of 95 °C for 20 s, 60 °C for 20 s, and 72 °C for 30 s, with a final extension at 72 °C for 10 min. Meanwhile, the first-strand cDNA and NS3 TA cloning primers were used to amplify an 832 bp NS3 fragment (GenBank accession number: KX977555.1) through PCR. The PCR reaction protocol consisted of an initial denaturation at 95 °C for 3 min, followed by 30 cycles of denaturation at 95 °C for 15 s, annealing at 60 °C for 15 s, and extension at 72 °C for 50 s, with a final extension at 72 °C for 5 min. The amplicons were cloned into the pCE2 TA/Blunt-Zero vector (Vazyme, Nanking, China). The copy concentration of the cloned plasmids was calculated based on their nucleic acid concentration and copy length using the online tool at https://www.technologynetworks.com/tn/tools/copynumbercalculator (accessed on 6 May 2024). The plasmids, diluted in a concentration gradient, were used to generate a standard curve through the aforementioned RT-qPCR, which was then employed to quantify the viral RNA concentration in the supernatant of cell culture media. Relative transcription levels were calculated using the ΔΔCT method as recommended by the manufacturer. The relative expression of target genes was normalized to the expression of the *GAPDH* gene.

### 2.9. Data Statistics

Each experimental group was set up with 3 biological replicates and 3 technical replicates. Data analysis and graphing were performed using GraphPad Prism 9.5 software. The comparison of differential gene expression between DTMUV-infected cells and uninfected control cells was conducted using a paired *t*-test, with a significance level of α = 0.05 (two-tailed).

## 3. Results

### 3.1. Validation of the DF-1 Cell Line as an Infection Model for DTMUV

**Figure 1:**
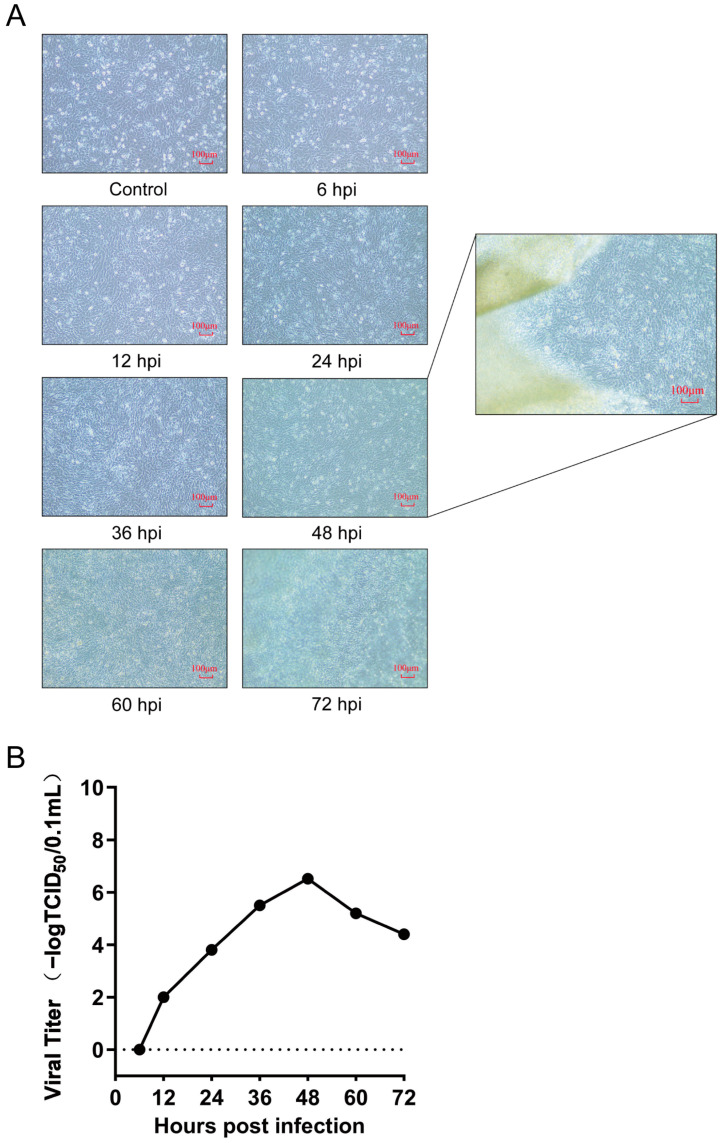
DTMUV infection in DF-1 cells. (**A**) The CPEs of DF-1 cells after DTMUV infection. The enlarged image on the right shows the field of view at the edge of the cell culture dish at 48 hpi, where some cells are seen to be detached in patches. ‘Control’ refers to DF-1 cells that were not inoculated with DTMUV, photographed at the 6th-hour post-inoculation (hpi) of the experimental group with DTMUV. ‘6 hpi’ denotes the cell status obtained 6 h after inoculation with 0.1 TCID_50_/cell of DTMUV, while the rest represent the cell statuses obtained at different hpi times in the experimental groups. (**B**) One-step growth curve of DTMUV in DF-1 cells.

The DF-1 cell line was validated as a reliable infection model for DTMUV through the examination of cytopathic effects (CPEs) and virus titer. Our results demonstrated that upon inoculation with DTMUV at a m.o.i. of 0.1 TCID_50_/cell, DF-1 cells began to exhibit mild CPEs at 24 hpi, with distinct CPEs visible at 48 hpi, characterized by regional detachment of DF-1 cells along the edges of the culture dish, where cells “lifted” and floated in the maintenance medium in a cohesive manner. Between 60 and 72 hpi, extensive detachment of cells was observed, with large clusters floating in the maintenance medium (Figure 1A). According to the one-step growth curve of DTMUV infection in DF-1 cells, the virus titer reached 2 logTCID_50_/0.1 mL at 12 hpi, subsequently increased to 6.52 logTCID_50_/0.1 mL at 48 hpi, and then declined (Figure 1B). The time point of high virus titer without significant cytoskeletal or membrane rearrangement in host cells is generally considered optimal for proteome analysis sample collection [28]. Therefore, we selected 48 hpi, with a high virus titer, as the infection condition for further proteome analysis.

### 3.2. Analysis of Differentially Expressed Proteins in Cells via 4D-DIA LC-MS/MS

To investigate the proteomic changes in DF-1 cells infected with DTMUV, protein extracts were collected from DTMUV-infected and mock-infected DF-1 cells at 48 hpi and preprocessed as described in Section 2.3. In total, 7624 unique proteins were detected and quantified. Among these, 157 differentially expressed proteins (DEPs) were identified based on fold change and *p*-value comparisons between DTMUV-infected samples and uninfected (mock) control samples. These included 84 significantly upregulated proteins (fold change > 1.5, *p* < 0.05; Supporting Information Table S2) and 73 significantly downregulated proteins (fold change ≤ 0.6667, *p* < 0.05; Supporting Information Table S2).

**Figure 2:**
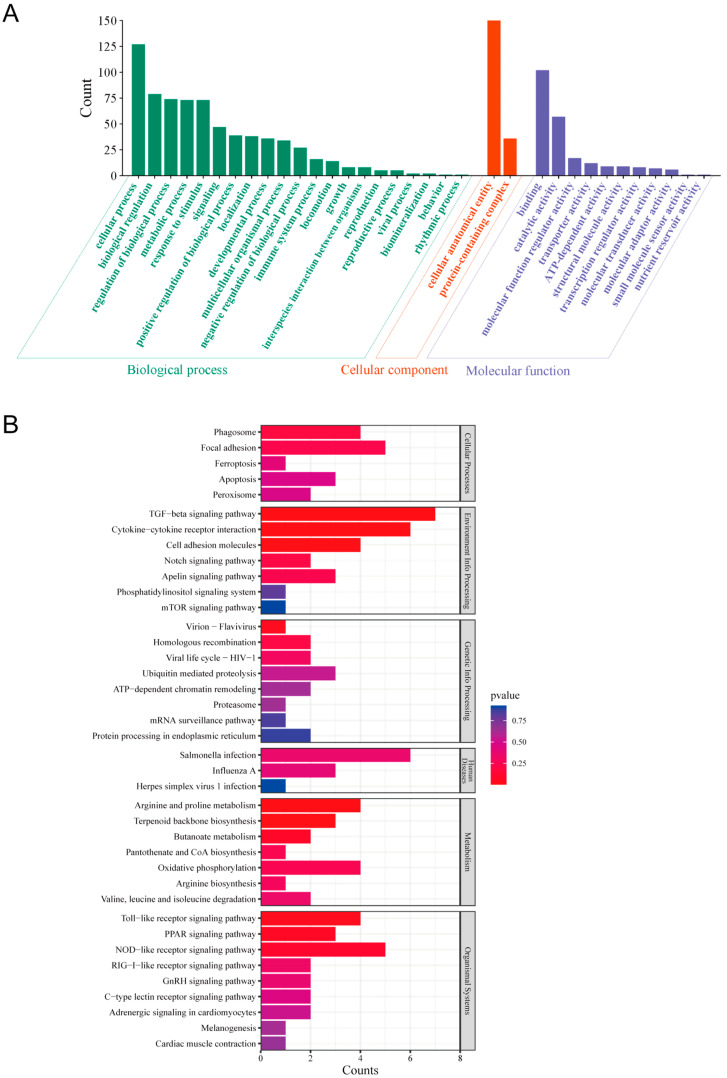
The GO functional analysis and KEGG pathway enrichment analysis were performed on the differentially expressed proteins (DEPs) in DF-1 cells infected with DTMUV. (**A**) GO annotation classification of differentially expressed proteins after 48 h of DTMUV infection of DF-1 cells. (**B**) KEGG pathway clustering of differentially expressed proteins 48 h after DTMUV infection of DF-1 cells.

All identified proteins were categorized into Gene Ontology (GO) molecular functional groups, revealing their involvement in 498 biological processes (with 192 processes having *p*-values < 0.05), 138 cellular components (with 27 components having *p*-values < 0.05), and 206 molecular functions (with 51 functions having *p*-values < 0.05). DTMUV infection was primarily associated with biological processes such as cellular metabolism, biological regulation of metabolism, stress response, and signal transduction. Furthermore, the majority of differentially expressed proteins (DEPs) were primarily localized to two cellular components: cellular anatomical entities and protein-containing complexes. In terms of molecular function annotations, DTMUV infection primarily impacted molecular binding, catalytic activity, and molecular function regulator activity (Figure 2A). Additionally, the identified DEPs were analyzed using the KEGG pathway database. A total of 77 metabolic pathways were enriched, with 9 pathways having *p*-values < 0.05. These pathways were grouped into six clusters: cellular processes, environmental information processing, genetic information processing, human diseases, metabolism, and organismal systems (Figure 2B). Notably, KEGG pathway enrichment analysis revealed high correlations between DTMUV infection and the TGF-β signaling pathway, cytokine-cytokine receptor interaction, NOD-like receptor signaling pathway, Toll-like receptor signaling pathway, and RIG-I-like receptor signaling pathway.

### 3.3. RT-qPCR Validation of Differentially Expressed Proteins

**Figure 3:**
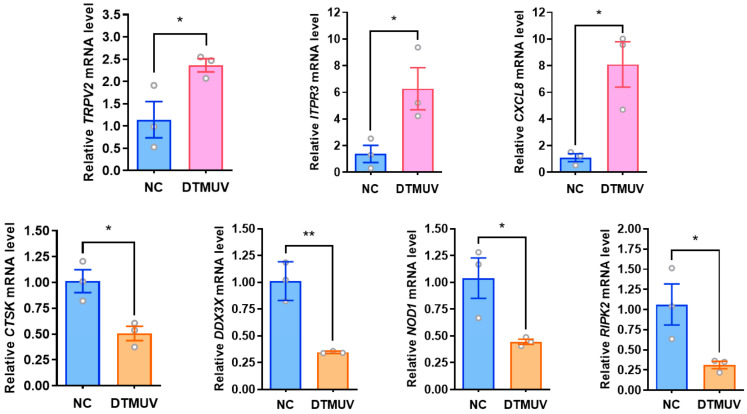
Validation of differentially expressed genes associated with the pattern recognition receptor pathway. Forty-eight hours after DTMUV infection of DF-1 cells, mRNA levels of PRR pathway-related genes were verified by fluorescence quantitative RT-qPCR, in which the Toll-like receptor signaling pathway had *CTSK* and *CXCL8*, the RIG-I-like receptor signaling pathway had *DDX3X* and *CXCL8*, whereas the NOD-like receptor signaling pathway had *RIPK2*, *ITPR3*, and *CXCL8*, *NOD1*, and *TRPV2*. *, *p* < 0.05; **, *p* < 0.01.

**Figure 4:**
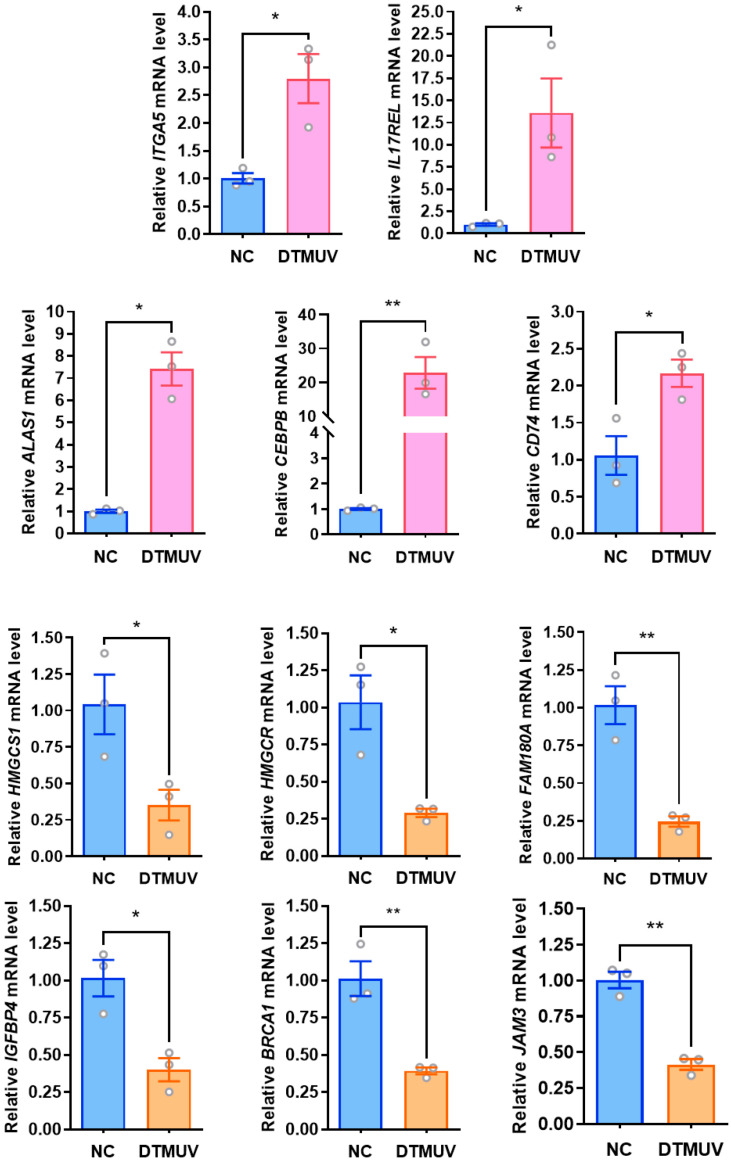
Validation of RT-qPCR on randomly selected differentially expressed genes. Forty-eight hours after DTMUV infection of DF-1 cells, randomly selected up-regulated as well as down-regulated differentially expressed genes were validated by fluorescence quantitative RT-qPCR. *, *p* < 0.05; **, *p* < 0.01.

To validate the changes in differentially expressed proteins (DEPs) identified by 4D-DIA quantitative proteomics at the transcriptional level, this study selected genes associated with three immune-related biological system-enriched pathways (Toll-like receptor signaling pathway: CTSK, CXCL8; RIG-I-like receptor signaling pathway: DDX3X, CXCL8; NOD-like receptor signaling pathway: RIPK2, ITPR3, CXCL8, NOD1, TRPV2) from DTMUV-infected DF-1 cells at 48 hpi, along with a subset of randomly selected DEPs (upregulated: CEBPB, CD74, ALAS1, ITGA5, and IL17REL; downregulated: HMGCS1, HMGCR, FAM180A, IGFBP4, BRCA1, and JAM3) and the DTMUV *NS3* gene. GAPDH was used as the internal reference gene for RT-qPCR validation. We found that all transcriptional trends were consistent with the 4D-DIA quantitative proteomics results, with mRNA expression of *CTSK*, *DDX3X*, *RIPK2*, and *NOD1* genes downregulated in pattern recognition receptor (PRR)-related pathways, while *TRPV2*, *ITPR3*, and *CXCL8* genes showed upregulated mRNA expression (Figure 3). The mRNA expression changes of some randomly selected DEPs were also consistent with the proteomic identifications (Figure 4).

### 3.4. TRPV2 Modulates DTMUV Replication in DF-1 Cells

**Figure 5:**
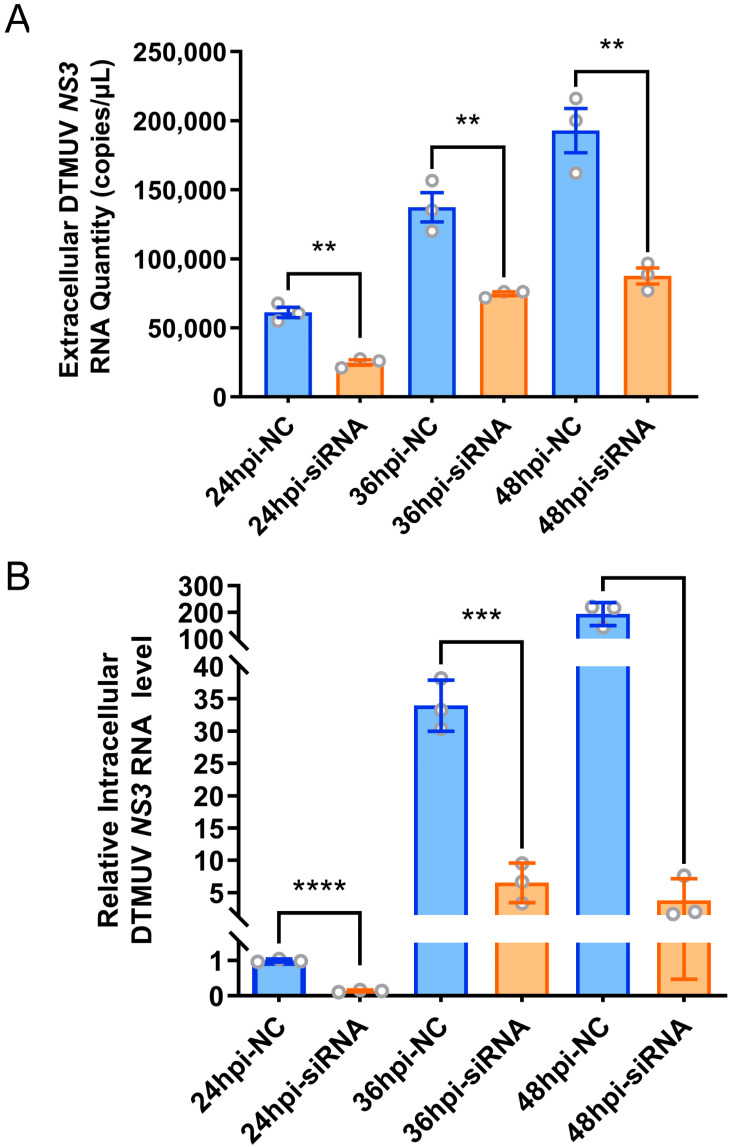
Effect of knockdown of TRPV2 on DTMUV replication. (**A**) Absolute quantification qPCR detection of the quantity of extracellular DTMUV *NS3* RNA; (**B**) RT-qPCR detection of *NS3* in cells gene relative expression. **, *p* < 0.01; ***, *p* < 0.001; ****, *p* < 0.0001.

Given the possibility that proteins localized on the cell membrane may serve as viral receptor proteins, and with the consideration of avoiding previously reported antiviral proteins against DTMUV, we prioritized Transient Receptor Potential Vanilloid 2 (TRPV2), a non-selective calcium ion channel protein located on the cell membrane and a member of the NOD-like receptor signaling pathway, as a preferred candidate antiviral host factor. Notably, TRPV2 was significantly upregulated in DF-1 cells infected with DTMUV at 48 hpi (Supporting Information Table S2). To investigate whether knocking down TRPV2 can inhibit DTMUV replication in DF-1 cells, we first needed to screen for an efficient TRPV2-specific siRNA. The results showed that all three designed siRNAs targeting the TRPV2 gene exhibited high gene silencing efficiency. However, TRPV2-siRNA-1 demonstrated the highest and most significant silencing efficiency (Supporting Information Figure S1). Therefore, we selected TRPV2-siRNA-1 as the most effective TRPV2 gene silencing siRNA and transfected it into DF-1 cells, using NC-siRNA as a control. Twenty-four hours post-transfection, the cells were infected with DTMUV at an equivalent m.o.i. of 0.1 TCID_50_/cell. Using RT-qPCR for absolute quantitation of the NS3 gene in the supernatants of cell culture media at different infection time points (24, 36, and 48 hpi), the obtained standard curve was y = −1.408ln(x) + 40.902 with an R^2^ of 0.9908 (where y represents the CT value, x denotes the quantity in copies/μL, and R^2^ is the coefficient of determination). The results showed that *TRPV2* knockdown significantly reduced the extracellular virus titer in DF-1 cells compared to control cells (Figure 5A). Additionally, relative RT-qPCR was employed to quantify NS3 gene expression within infected cells at 24, 36, and 48 hpi. The results showed that, compared to the control cells, *TRPV2* knockdown also significantly decreased intracellular viral replication in DF-1 cells (Figure 5B).

## 4. Discussion

As an emerging member of the Orthoflavivirus within the Flaviviridae family, duck Tembusu virus (DTMUV) has inflicted substantial economic losses on the poultry industry in Eastern and Southeast Asia. Furthermore, due to the scarcity of specific antiviral drugs, the development of vaccines against DTMUV has become an urgent task for preventing and controlling its spread. In the pursuit of attenuated vaccine candidates, continuous passaging of the virus in susceptible cells is commonly employed to attenuate its virulence [11,29]. Given their simplicity and cost-effectiveness, DF-1 cells are routinely utilized for the production of avian vaccines [30]. It is noteworthy that although DTMUV primarily targets ducks, in recent years, variant strains of DTMUV have emerged as a growing threat to chicken populations. A case in point is the DTMUV outbreak reported in chickens in Guangdong Province in 2020 [31]. Additionally, a variant strain of DTMUV isolated from White Roman geese in Taiwan at the end of that year demonstrated the capacity for direct contact transmission within chicken flocks, leading to pathological changes such as hepatomegaly and splenomegaly in chicks [1]. In comparison to primary duck embryo fibroblasts, which are limited in their passaging abilities and difficult to obtain, DF-1 cells offer distinct advantages, including stability, the absence of tumorigenic genes, and the capability for indefinite proliferation. These unique properties position DF-1 cells as the preferred research model for avian viruses [32,33]. Furthermore, DTMUV has been shown to exhibit robust proliferation in DF-1 cells [11,34]. Consequently, we have established DF-1 cells as the chosen platform for studying DTMUV infections. However, the underlying interaction mechanisms between DTMUV and DF-1 cells remain elusive, and the pathogenesis and immune regulatory mechanisms of DTMUV are yet to be fully elucidated. Quantitative proteomics, a powerful tool for high-throughput protein identification, has been widely applied to unravel virus–host interactions, offering a robust research strategy for uncovering potential antiviral factors in hosts. In this study, we employed 4D-DIA-based quantitative proteomics coupled with LC-MS/MS to analyze protein expression in DF-1 cells infected with DTMUV for 48 h. Consequently, 157 differentially expressed proteins (DEPs) were identified, including 84 upregulated and 73 downregulated proteins. Notably, DTMUV infection elicited multifaceted alterations in protein expression within the Toll-like, RIG-I-like, and NOD-like receptor signaling pathways of the innate immune system in DF-1 cells. Specifically, CTSK, RIPK2, NOD1, and DDX3X expression were significantly downregulated, whereas CXCL8, ITPR3, and TRPV2 expression were markedly upregulated. Consistent with our findings, Xiang et al. [35] utilized transcriptomics to reveal the activation of pattern recognition receptor pathways, including Toll-like, NOD-like, and RIG-I-like receptor signaling, in DTMUV-infected duck embryo fibroblasts (DEFs), further emphasizing the significance of these pathways in DTMUV infection. Furthermore, GO enrichment and KEGG pathway analyses were performed to explore the DEPs. The results indicated that DTMUV-induced DEPs in DF-1 cells were primarily involved in biological regulatory metabolism, immune regulatory metabolism, cellular responses to stimuli, and signal transduction processes. This discovery provides valuable insights into the intricate interplay between DTMUV and DF-1 cells, facilitating a deeper understanding of the underlying mechanisms governing their interactions.

To validate the accuracy of the proteomic identifications from 4D-DIA proteomics, we employed RT-qPCR to examine the mRNA levels of differentially expressed proteins related to pattern recognition receptors and randomly selected differentially expressed proteins in DTMUV-infected DF-1 cells at 48 h post-infection. Our findings concurred with the proteomic identification results. To further enhance the credibility of these findings and provide a solid foundation for understanding the interaction mechanisms between DTMUV and DF-1 cells, an in-depth analysis of representative differentially expressed proteins is necessary. Interleukin-8 (CXCL8), also known as IL-8, is a chemokine belonging to the CXC family, which is ubiquitously expressed in various cell types, including leukocytes, fibroblasts, epithelial cells, and hepatocytes. Typically, CXCL8 is expressed at low levels within cells, but its expression and secretion significantly increase in response to various external stimuli, such as bacteria, viruses and their products, hypoxia or hyperoxia, and reperfusion injury. In this study, CXCL8 protein was significantly upregulated in DF-1 cells 48 h after DTMUV infection, with the highest fold change in expression (Table S2), indicating that DTMUV infection induces CXCL8 expression in DF-1 cells, thereby alerting the host immune system to viral infection. Similarly, Li et al. [36] observed upregulated *CXCL8* gene expression in the spleen and brain of 1-day-old ducklings infected with DTMUV. Moreover, DDX3X, a member of the DEAD-box RNA helicase family and the RIG-I-like receptor signaling pathway, is localized in the cytoplasm or nucleus and plays crucial roles in RNA metabolism, innate immune signaling, and cell cycle regulation. Given that RNA metabolism is a target for RNA viruses that rely entirely on host cell machinery for proliferation, DDX3X helicase has been described as a host factor influencing viral replication and a cellular sensor inducing innate immune responses. Studies have reported the inhibitory effect of DDX3X on DTMUV replication, with overexpressed duck-derived DDX3X positively regulating type I interferon in DEFs and suppressing DTMUV replication during early infection, although it did not significantly impact the expression of pro-inflammatory cytokines such as IL-1β, IL-6, and CXCL-8 [37]. In our study, DTMUV infection induced downregulation of DDX3X protein, suggesting that DDX3X may act as an inhibitor of DTMUV infection. Consistently, Sun et al. [38] used iTRAQ proteomics to identify significant downregulation of DDX3X in DTMUV-infected hamster kidney fibroblasts (BHK-21) and demonstrated that DDX3X inhibits DTMUV replication via the TBK1-mediated interferon pathway. Furthermore, our study found that the mRNA level of DDX3X was also significantly downregulated in DF-1 cells 48 h after DTMUV infection, and multiple studies have shown that DDX3X inhibits DTMUV replication in both BHK-21 and DEF cells. Thus, this study does not delve further into the inhibitory effects of DDX3X on DTMUV replication.

This study validated the genes enriched in the Toll, RIG-I, and NOD-like receptor signaling pathways identified by KEGG analysis, and the differential mRNA expression results of *CTSK*, *CXCL8*, *DDX3X*, *RIPK2*, *ITPR3*, *NOD1*, and *TRPV2* concurred with the proteomics identification. These genes can be considered as candidate genes for resistance against DTMUV infection. Given the possibility that membrane-bound proteins may serve as viral receptors and to avoid previously reported antiviral proteins against DTMUV, Transient Receptor Potential Vanilloid 2 (TRPV2), a member of the NOD-like receptor signaling pathway and a non-selective calcium ion channel protein localized on the cell membrane, was selected as a preferred candidate host factor for disease resistance. The significant upregulation of TRPV2 expression in DF-1 cells 48 h post-DTMUV infection (Supporting Information Table S2) indicates its potential role in facilitating viral infection during DTMUV entry into avian cells. Recent research suggests that TRPV2 promotes cell membrane tension and fluidity via the calcium-LRMDA axis in myeloid cells, thereby facilitating viral penetration. Knocking out TRPV2 or inhibiting its channel activity in myeloid cells inhibits viral infection and protects mice from Herpes Simplex Virus type 1 (HSV-1) and Vesicular Stomatitis Virus (VSV) [39]. Based on these findings, we hypothesize that TRPV2 is a host factor necessary for DTMUV replication. To validate this hypothesis, we identified an efficient siRNA that knocks down *TRPV2* gene expression and further investigated its impact on DTMUV replication in DF-1 cells. We found that knocking down the expression of *TRPV2* in DF-1 cells inhibited intracellular DTMUV replication and decreased extracellular virus titer, suggesting that TRPV2 is a potential host factor in poultry against DTMUV infection. This target can be leveraged in future disease-resistant breeding programs to develop poultry individuals or populations resistant to DTMUV. It is worth mentioning that in our research exploring the interaction between DTMUV and poultry hosts, we primarily focused on uncovering host resistance factors and further investigating the mechanisms by which these host factors induce immune responses. Zhang et al. [40] reported that the non-structural protein 2A (NS2) of DTMUV binds to duck STING (stimulator of interferon genes) in DEFs, thereby disrupting the induction of interferon signaling cascades and inhibiting beta interferon induction. This finding underscores the virus’s ability to interfere with the host’s antiviral response. In contrast, while Wei Zhang et al. provided insights into viral proteins inhibiting interferon signaling, our work complements this by proposing host factors TRPV2 that may promote viral replication. Furthermore, the utilization of different cell viral infection models (DEFs versus DF-1 cells) and the shared focus on proteomic alterations collectively provide a more comprehensive perspective for understanding the interactions between the host and virus during DTMUV infection. 

## 5. Conclusions

In summary, this study is the first attempt to identify proteomic changes in DF-1 cells infected with DTMUV using a quantitative proteomics approach based on 4D-DIA. Among the altered proteins, we noted the upregulation of the pattern recognition receptor TRPV2. Our findings reveal that DTMUV replication is inhibited in DF-1 cells with low TRPV2 expression, suggesting that TRPV2 may be an essential host factor for DTMUV infection. Furthermore, in-depth analysis and functional studies of these differentially expressed proteins will facilitate the screening and identification of host factors against DTMUV, thereby providing targets and evidence for the development of disease-resistant poultry in animal breeding programs.

## Figures and Tables

**Table 1:** Primers for RT-qPCR.

Names	Accession No.	Forward Primer Sequence (5′→3′)	Reverse Primer Sequence (5′→3′)
NS3-TAclone	KX977555.1	GAGGCTCACTTCACAGACCC	ACCGCCGGTCATTGTAACTT
NS3	HQ641389.1	TTCATGACAGCCACACCTCC	GTCAAGCACACGGCAATCTC
GAPDH	NM_204305.2	GTCCATGCCATCACAGCCACAC	CATCAGCAGCAGCCTTCACTACC
DDX3X	NM_001030800.2	ATGAGGAGGCCAGGAAGTTT	CTGAGGTTCGAAACCCATGT
NOD1	NM_001318438.1	GCGATGCAGGAATTGGAAAA	TGTGAAAAGAACCGTATGAGGGA
RIPK2	NM_001030943.2	CTCGAACCAGTCCTGAGAACG	AAGCGGATGTTTCCTCTTGG
HMGCS1	NM_205411.1	CAGTTCTTGGGATGGACGCT	CCCAACTAGCATAGCAACAGC
CTSK	XM_046903916.1	CATCATTGACGGAGCGATGC	TTTCGTCCTCCTTGCCGTTG
HMGCR	NM_204485.1	TTGGATAGAGGGAAGAGGGAAG	CCATAGCAGAACCCACCAGA
FAM180A	JN032743.1	GACTTTGCTGCTGCTGCTGTTC	CTCAGCAGCCTTCCTCAGCGA
IGFBP4	NM_204353.1	AACTTCCACCCCAAGCAG	AATCCAAGTCCCCCTTCAG
BRCA1	NM_204169.1	AAGCCGATTGTGAGCGAAGA	CCATTCCGATCAGCGAGTGA
JAM3	XM_417876.6	CCAGAGTGTTGAGCTGTCCT	AGAATTTCTGCCCGAGTTGC
TRPV2	XM_040650471.2	CAGTCAGGCCCATGTCAGAG	GGTAAGGTGACTCCAAGGGC
CXCL8	AJ009800.1	CCAAGCACACCTCTCTTCCA	GCAAGGTAGGACGCTGGTAA
ITPR3	XM_015298988.4	TGTTCCGACTGTGCTACCG	AAGGCTGTGATGGTGTCCTC
CEBPB	NM_205253.3	TTTGCTTTCATGCAACGCCT	TAAGTTCGGTCATGGAGCGG
CD74	NM_001001613.2	CAGTTTCGGTGGTGTCCATC	GTCCACCCCAGAGAAGATCAC
ALAS1	NM_001018012.2	CATCTCTGGAACGCTCGGCAAG	CCAGCAGCATACGAACGGACAG
ITGA5	XM_046904986.1	CTCCAACTACCCCGAGTACT	CACCGAATAGCCCATATAAC
IL17REL	XM_015273696.4	TCCAGATGCCAGGAGGTTAC	TGCAAAGTCTTGGGTGAGTG

## Data Availability

The mass spectrometry proteomics data have been deposited to the ProteomeXchange Consortium (https://proteomecentral.proteomexchange.org accessed on 2 September 2024) via the iProX partner repository with the dataset identifier PXD055452.

## Supplementary Materials

The following supporting information can be downloaded at: https://www.mdpi.com/article/10.3390/v16121831/s1, Table S1: *TRPV2* siRNA sequences; Table S2: Differentially expressed proteins in DF-1 cells after 48 h of DTMUV infection, Figure S1: Screening of efficient siRNAs for silencing the chicken *TRPV2* gene.
