# Supplementary material for: 4D-DIA-Based Quantitative Proteomic Analysis Reveals the Involvement of TRPV2 Protein in Duck Tembusu Virus Replication
Source: Viruses. 2024 Nov 26;16(12):1831. doi: 10.3390/v16121831 (PMC11680370; doi:10.3390/v16121831)
Supplement: Supplementary file 1 [file viruses-16-01831-s001.zip › viruses-3289811-supplementary.pdf]

Table S1. *TRPV2* siRNA sequences

siRNA	Accession no.	Forward primer sequence (5'→3')	Reverse primer sequence (5'→3')
NC-siRNA	XM_040650471.2	ACGUGACACGUUCGGAGAATT	UUCUCCGAACGUGUCACGUTT
TRPV2-siRNA-1		ACUAGAACUUUCACUAAGUCCTT	GGACUUAGUGAAAGUUCUAGUTT
TRPV2-siRNA-2		UUUCUAGCAGUCCUACCAACCTT	GGUUGGUAGGACUGCUAGAAATT
TRPV2-siRNA-3		UAUUGUCCUGAUCUUUCUUCCTT	GGAAGAAAGAUCAGGACAAUATT

Table S2. Differentially expressed proteins in DF-1 cells after 48h of DTMUV infection

Proteins description	Accession on.	Fold change (infection/control)	P-value (infection/control)	Regulation
Transient receptor potential cation channel subfamily V member 2	A0A3Q2UBT8	1.504156582	0.00896459	up
Carboxymethylenebutenolidase homolog	A0A8V0X6C8	1.506191794	0.00726137	up
Ubiquitin-like protein	A0A8V0XRY6	1.509803824	0.04007591	up
Metalloproteinase inhibitor 2	O42146	1.512176637	0.01357385	up
HECT-type E3 ubiquitin transferase	A0A8V0ZKK3	1.515364228	0.00558371	up
Membrane associated guanylate kinase, WW and PDZ domain containing 2	A0A3Q2UEI2	1.516071906	0.01975816	up
Cytochrome c oxidase copper chaperone COX11	A0A8V1AL15	1.517157775	0.03620451	up
Tubulin-specific chaperone A	A0A8V0YFY8	1.521005267	0.01390197	up
NHR domain-containing protein	A0A8V0X4G8	1.522568483	0.02737096	up
RNA 3'-terminal-phosphate cyclase (ATP)	A0A8V0ZP45	1.522878152	0.04945085	up
ATP synthase subunit e, mitochondrial	Q9YH14	1.527213666	0.04320621	up
Cilia and flagella associated protein 43	A0A8V0ZC57	1.52954628	0.03793049	up
Sorting nexin 10	A0A8V0X400	1.549021994	0.03885975	up
Multivesicular body subunit 12A	Q5ZJX7	1.552129025	0.03146112	up
NADH dehydrogenase [ubiquinone] 1 beta subcomplex subunit 4	A0A8V1A181	1.561520885	0.01462031	up
Integrin subunit alpha 5	A0A8V0ZI40	1.567257115	0.00133023	up
Aminoacylase 1	A0A8V1AEU7	1.576949751	0.04387422	up
Interleukin 17 receptor E like	A0A8V0XVL9	1.580082984	0.01652355	up
Uncharacterized protein	A0A8V0Z4G6	1.583251195	0.00303469	up
Family with sequence similarity 76 member B	A0A8V0XTF1	1.583308394	0.04820938	up
Thrombospondin 1	A0A8V0ZHF5	1.583735436	0.00927160	up
Decorin	P28675	1.602890037	0.03170438	up
Protocadherin 19	A0A8V0YT49	1.608629667	0.00600880	up
cAMP responsive element binding protein 3 like 2	F1P1J2	1.617057661	0.01185185	up

Table S2. Differentially expressed proteins in DF-1 cells after 48h of DTMUV infection

Proteins description	Accession on.	Fold change (infection/control)	P-value (infection/control)	Regulation
Insulin-like growth factor-binding protein 5	F1ND88	1.622937688	0.03916287	up
Nucleic acid binding protein 2	A0A8V1A0N3	1.630443646	0.00377719	up
Dual specificity phosphatase 10	F1NXH3	1.634884374	0.02152036	up
Latent transforming growth factor beta binding protein 1	A0A8V0XTQ7	1.650206125	0.01719975	up
STEAP4 metalloreductase	A0A8V0XHA5	1.652217314	0.00289853	up
Cytohesin 1	A0A8V1AH87	1.657485996	0.02528232	up
CCN family member 1	P19336	1.666016752	0.00012833	up
Tropomyosin 1	A0A8V0ZYD6	1.666308052	0.03442187	up
Bardet-Biedl syndrome 5	A0A8V0ZXY6	1.667493551	0.00008026	up
Tsukushi	Q65Z91	1.667736379	0.03679082	up
Vitellogenin 3	A0A8V0ZF17	1.69313777	0.00512225	up
Small nuclear ribonucleoprotein G	E1C8V9	1.700482696	0.02382411	up
T-box 3	A0A1D5NXF3	1.720420014	0.02672245	up
Chloride intracellular channel protein	A0A8V0YT21	1.724676842	0.00209866	up
Uncharacterized protein	A0A8V1AFD3	1.74418001	0.03310701	up
GrpE like 2, mitochondrial	A0A8V0YCG1	1.747519468	0.03386007	up
RAS like proto-oncogene A	A0A1D5P1T0	1.763832299	0.01097172	up
CD81 molecule	A0A8V0ZY93	1.764464285	0.03662957	up
Periostin	A0A3Q2U321	1.793081125	0.01437532	up
Renin receptor	A0A8V0XAH4	1.844818392	0.01974311	up
Bactericidal permeability-increasing protein	A0A8V0YR09	1.853295176	0.04351912	up
Inhibin beta A chain	P27092	1.890289967	0.01408457	up
Ubiquitin conjugating enzyme E2 Q2	A0A8V0ZZY2	1.892584258	0.04333672	up
Ring finger protein 24	R4GLL9	1.8996139	0.04792133	up
Protein-L-isoaspartate O-methyltransferase domain-containing protein 1	Q5ZMR3	1.907116093	0.01962347	up
Apolipoprotein B	A0A8V0XV56	1.908651141	0.00595566	up
Programmed cell death protein 4	Q98TX3	1.911078368	0.00072203	up

Table S2. Differentially expressed proteins in DF-1 cells after 48h of DTMUV infection

Proteins description	Accession on.	Fold change (infection/control)	P-value (infection/control)	Regulation
Choline/carnitine acyltransferase domain-containing protein	A0A8V0XIT0	1.912864352	0.00535557	up
T-complex 11 family, X-linked 2	A0A8V0YF55	1.920725905	0.00846288	up
Bromodomain containing 7	A0A8V0ZQG1	1.930478956	0.01463525	up
Fibronectin	P11722	1.941255883	0.03649039	up
Mitogen-activated protein kinase 6	Q5F3W3	1.961911348	0.00978772	up
5-aminolevulinate synthase, non-specific, mitochondrial	P07997	1.966418937	0.01538084	up
CD74 molecule	A0A8V0YKP1	1.986247131	0.01388042	up
Unc-13 homolog D	A0A8V1AFD5	1.993179057	0.00331167	up
Vascular endothelial growth factor receptor 1	Q8QHL3	2.025134588	0.03107951	up
Glucosamine-6-phosphate isomerase	A0A8V0YL49	2.034299888	0.03784039	up
Complement C1s	A0A8V0ZX35	2.072340672	0.00032812	up
Latent transforming growth factor beta binding protein 1	A0A8V0XVP9	2.083832233	0.01332047	up
CN hydrolase domain-containing protein	A0A8V0XTE5	2.093105987	0.00577520	up
Metalloproteinase inhibitor 3	P26652	2.121944162	0.00735868	up
Uncharacterized protein	A0A8V0YLK4	2.209160461	0.01893701	up
E3 ubiquitin-protein ligase	A0A3Q3A808	2.229735616	0.02199262	up
CCAAT/enhancer-binding protein beta	Q05826	2.24742142	0.00027952	up
Pleckstrin homology like domain family A member 1	A0A8V0YNC7	2.304130602	0.04824754	up
E3 ubiquitin-protein ligase	E1BWT2	2.384197274	0.00025955	up
KIAA1671	A0A8V0ZP50	2.56693851	0.02273645	up
Inositol 1,4,5-trisphosphate receptor	A0A8V1ACE6	2.636803435	0.04256423	up
NFKB inhibitor zeta	A0A8V0XKE6	2.694883975	0.00028834	up
Cellular communication network factor 2	Q98TQ8	2.731327465	0.00000913	up
Inter-alpha-trypsin inhibitor heavy chain 3	A0A8V1A774	2.745414381	0.00068876	up
CCHC-type domain-containing protein	A0A3Q2TV99	2.824221628	0.00180127	up
mRNA decay activator protein ZFP36	S5TQ07	2.856194233	0.00281063	up

Table S2. Differentially expressed proteins in DF-1 cells after 48h of DTMUV infection

Proteins description	Accession on.	Fold change (infection/control)	P-value (infection/control)	Regulation
SUN domain-containing protein	A0A8V1ADV7	2.951319795	0.00756721	up
Angiopoietin like 4	A0A8V1AHP7	3.255067218	0.03400966	up
DNA repair protein	A0A1D5NTZ0	3.313136948	0.02352408	up
Keratin, type I cytoskeletal 19	O93256	3.34739039	0.04458394	up
Syndecan-4	P49416	3.35389818	0.01796646	up
39S ribosomal protein L35, mitochondrial	A0A8V0ZAL3	3.375980857	0.04073420	up
Interleukin-8	P08317	8.472024977	0.00080680	up
Receptor-interacting serine/threonine-protein kinase 2	A0A8V0YED1	0.110744335	0.00004771	down
NOD1	A0A1D5PTE0	0.278774019	0.00064264	down
FHF complex subunit HOOK interacting protein 2A	A0A8V0ZID6	0.373933661	0.02074708	down
Ring finger protein 169	A0A8V0X3U0	0.41624957	0.04121121	down
C1q and TNF related 3	R4GKL8	0.433687857	0.00064346	down
Regulator of G protein signaling 1	A0A8V0YNR5	0.441758851	0.04751762	down
Podocan	A0A8V1A5Z6	0.449865013	0.02887682	down
Integrin beta 1 binding protein 3	A0A8V0YT25	0.469810478	0.02676777	down
Leucine rich repeats and immunoglobulin like domains 1	A0A8V0Z9G8	0.470277353	0.04937306	down
Pleckstrin 2	A0A8V0ZBA3	0.480477436	0.00016271	down
Actin filament associated protein 1 like 2	A0A8V0ZHY8	0.48199859	0.00084619	down
Oligophrenin 1	A0A8V0YHG8	0.510673439	0.01606947	down
RNA binding motif protein 34	A0A8V0Y6A1	0.522631565	0.00287981	down
Cathepsin K （PE=2）	Q90686	0.539674412	0.03046623	down
Serine/threonine kinase 17b	Q5ZM34	0.540375283	0.00343316	down
Zinc finger protein 703	A0A8V0Z9N7	0.541095526	0.00646825	down
Mitochondrial ribosomal protein L54	A0A8V1A7B7	0.54543379	0.01469943	down
trans-L-3-hydroxyproline dehydratase	A0A8V0Z6G7	0.545708683	0.03202359	down
3-hydroxy-3-methylglutaryl coenzyme A reductase	A0A8V0Y352	0.558378487	0.00480545	down
Zinc and ring finger 2	A0A8V0YYC5	0.564548595	0.04766670	down

Table S2. Differentially expressed proteins in DF-1 cells after 48h of DTMUV infection

Proteins description	Accession on.	Fold change (infection/control)	P-value (infection/control)	Regulation
Family with sequence similarity 180 member A	A0A8V0Y365	0.569300158	0.00465320	down
CD99 molecule (human Xg blood group)	A0A8V0X907	0.572328103	0.03851640	down
Lumican	P51890	0.574929165	0.03070243	down
DNA repair protein SWI5 homolog	A0A8V1AF36	0.576517945	0.03720965	down
Ubiquitin like 5	A0A1D5PWV5	0.581079456	0.02372404	down
Nuclear factor 1 A-type	P17923	0.583252595	0.02708137	down
Multiple inositol polyphosphate phosphatase 1	F1NPQ2	0.584334246	0.00715248	down
ADP-ribosylation factor-like protein 2-binding protein	Q5ZKW5	0.588594521	0.04761553	down
Tumor necrosis factor alpha-induced protein 8	Q5ZI78	0.589733918	0.02492681	down
Proline rich coiled-coil 2B	A0A8V1AFE0	0.593988409	0.04672822	down
Poly [ADP-ribose] polymerase	A0A8V0XF27	0.597250978	0.02597331	down
Patatin like phospholipase domain containing 6	A0A1D5PGA8	0.598047888	0.03358236	down
Biogenesis of lysosome-related organelles complex 1 subunit 6	A0A8V0YSV9	0.607212212	0.02758841	down
Cytidine and dCMP deaminase domain containing 1	A0A1D5NZE1	0.608599093	0.04914165	down
Mitogen-activated protein kinase kinase 6	A0A1D5PHN6	0.608668712	0.01881699	down
Zinc finger protein 827	A0A8V0XLH7	0.611075851	0.01264206	down
Uncharacterized protein	A0A8V0ZD15	0.61214424	0.01505528	down
UBZ1-type domain-containing protein	A0A8V1A0N6	0.612435318	0.00396343	down
Cathepsin K （PE=3）	A0A8V1AA62	0.613715854	0.04772629	down
Hydroxymethylglutaryl-CoA synthase	A0A1L1RZ64	0.615713217	0.03459228	down
Actin binding LIM protein 1	A0A8V0ZNG3	0.615907791	0.00470677	down
BRCA1 DNA repair associated	A0A8V1A3P9	0.619898479	0.01199786	down
Junctional adhesion molecule 3	A0A8V0ZX45	0.621373244	0.00097946	down
tryptophan 5-monooxygenase	A0A8V0YAT6	0.624333362	0.03415882	down
Interleukin 6 signal transducer	A0A8V0YBA0	0.626872414	0.04498245	down
Actin filament associated protein 1 like 2	A0A8V0ZHZ4	0.628277393	0.00841157	down
Transcription factor 12	A0A8V0ZRR8	0.632452524	0.00868908	down

Table S2. Differentially expressed proteins in DF-1 cells after 48h of DTMUV infection

Proteins description	Accession on.	Fold change (infection/control)	P-value (infection/control)	Regulation
ALX homeobox 4	O93582	0.634736451	0.03176689	down
Pre-mRNA 3'-end-processing factor FIP1	A0A8V0YH15	0.638732498	0.04867546	down
Ectodysplasin A2 receptor	A0A8V0YRL1	0.638738825	0.02999353	down
PHD finger protein 20	A0A8V0ZFL3	0.640806451	0.04156469	down
Proteasome 26S subunit, non-ATPase 14	A0A8V0ZG26	0.640893876	0.00997962	down
Iduronate 2-sulfatase	A0A1L1RU31	0.642725577	0.01611603	down
Collagen type VIII alpha 1 chain	E1C353	0.647033791	0.03758367	down
DEAD-box helicase 3, X-linked	A0A3Q2U0S7	0.649186094	0.00533292	down
Regulatory factor X associated ankyrin containing protein	A0A8V1AKE2	0.649317547	0.00518369	down
Starch binding domain 1	R4GJM5	0.649916574	0.00783824	down
Insulin-like growth factor-binding protein 4	A0A1D5P480	0.650070059	0.00792263	down
DGCR8, microprocessor complex subunit	A0A1D5PPX8	0.650975561	0.00993110	down
Peroxisomal biosis factor 5	A0A8V0ZP66	0.654578591	0.04666899	down
SIN3-HDAC complex-associated factor	Q5ZJV7	0.655137682	0.01271921	down
GULP PTB domain containing engulfment adaptor 1	A0A8V0YRB4	0.655959626	0.01024909	down
Lipid droplet-associated hydrolase	Q5F477	0.65691903	0.00404336	down
Cyclic AMP-dependent transcription factor ATF-7	A0A1D5PF49	0.658073674	0.03558941	down
RAS p21 protein activator 3	A0A8V0YH79	0.658467693	0.00873024	down
Fidgetin like 1	A0A8V0X8J6	0.659401202	0.00160686	down
Uncharacterized protein	A0A8V0X8W1	0.659492218	0.00153627	down
SR-related CTD associated factor 8	A0A8V0XG74	0.659965247	0.01607075	down
Signal peptide peptidase-like 2B	Q5F383	0.660072065	0.01139013	down
Hydroxymethylglutaryl-CoA synthase, cytoplasmic	P23228	0.662065724	0.00314330	down
Ankyrin repeat domain 40	A0A8V1ALE9	0.663323271	0.03069791	down
ADP-ribosylation factor-like protein 6	A0A1D5PWK2	0.665041002	0.02307605	down
Matrix remodeling-associated protein 8	Q90WI4	0.666081027	0.02908670	down

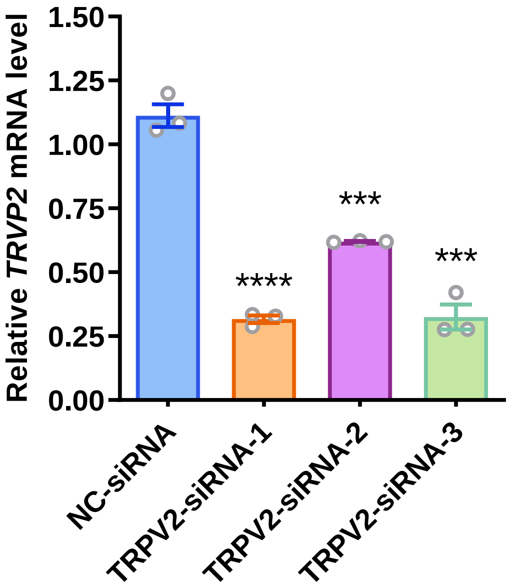

Figure S1. Screening of efficient siRNAs for silencing the chicken *TRPV2* gene. NC-siRNA-transfected DF-1 cells were used as a negative control group, and the expression level of *TRPV2* gene in the cells was detected using fluorescence quantitative RT-qPCR to identify the most efficient siRNA. ***, $p < 0.001$; ****, $p < 0.0001$.
